# Supplementary material for: Amygdala TDP-43 pathology is associated with behavioural dysfunction and ferritin accumulation in amyotrophic lateral sclerosis
Source: Brain Commun. 2026 Mar 20;8(2):fcag104. doi: 10.1093/braincomms/fcag104 (PMC13056708; doi:10.1093/braincomms/fcag104)
Supplement: fcag104_Supplementary_Data [file fcag104_supplementary_data.docx]

***Supplementary table 1. Cohort demographics.***

Table detailing cohort demographics including Tau and TDP-43 severity scoring, clinical and cognitive data.

| **BB ID** | **Cell type** | **Tau IHC** | **pTDP-43 IHC** | | | | | | **Sex** | **Age at onset** | **Survival (months)** | **Cognitive assessment** | |
| --- | --- | --- | --- | --- | --- | --- | --- | --- | --- | --- | --- | --- | --- |
|  |  | **Amygdala** | **Amygdala** | **BA11/12** | **BA24** | **BA6** | **BA46** | **BA9** |  |  |  |  |  |
|  |  |  |  |  |  |  |  |  |  |  |  | **Behavioural deficit on ECAS** | **Other cognitive deficit on ECAS** |
| **Case 1** | Neuronal | 2 | 2 | 1 | 1 | 2 | 2 | 1 | M | 55 | 134 | Behavioural assessment not done | No |
| ARTAG staining pattern | Glial | 3 | 2 | 1 | 1 | 2 | 2 | 1 |  |  |  |  |  |
| **Case 2** | Neuronal | 2 | 1 | 1 | 1 | 1 | 1 | 0 | F | 65 | 130 | Behavioural assessment not done | Executive, language and fluency |
|  | Glial | 0 | 1 | 1 | 1 | 1 | 1 | 0 |  |  |  |  |  |
| **Case 3** | Neuronal | 2 | 0 | 0 | 0 | 1 | 0 | 1 | F | 58 | 99 | Behavioural assessment not done | Fluency |
|  | Glial | 0 | 0 | 0 | 1 | 2 | 1 | 1 |  |  |  |  |  |
| **Case 4** | Neuronal | 1 | 3 | 3 | 3 | 3 | 3 | 3 | M | 53 | 94 | Yes | ECAS not done |
|  | Glial | 0 | 2 | 3 | 2 | 2 | 3 | 3 |  |  |  |  |  |
| **Case 5** | Neuronal | 3 | 1 | 0 | 1 | 1 | 0 | 0 | F | 66 | 24 | Yes | No |
|  | Glial | 0 | 1 | 0 | 1 | 1 | 1 | 0 |  |  |  |  |  |
| **Case 6** | Neuronal | 1 | 3 | 3 | 2 | 3 | 3 | 3 | M | 56 | 14 | Behavioural assessment not done | Fluency |
|  | Glial | 0 | 2 | 2 | 2 | 3 | 3 | 2 |  |  |  |  |  |
| **Case 7** | Neuronal | 0 | 2 | 2 | 2 | 2 | 1 | 1 | M | 55 | 20 | Behavioural assessment not done | Executive, language and fluency |
|  | Glial | 0 | 1 | 2 | 2 | 2 | 2 | 2 |  |  |  |  |  |
| **Case 8** | Neuronal | 2 | 3 | 1 | 1 | 0 | 1 | 1 | M | 89 | 14 | Behavioural assessment not done | ECAS not done |
|  | Glial | 3 | 1 | 1 | 0 | 2 | 1 | 1 |  |  |  |  |  |
| **Case 9** | Neuronal | 1 | 0 | 0 | 1 | 2 | 1 | 0 | F | 73 | 98 | No | Executive dysfunction |
|  | Glial | 0 | 0 | 0 | 0 | 1 | 1 | 0 |  |  |  |  |  |
| **Case 10** | Neuronal | 0 | 0 | 0 | 0 | 2 | 1 | 1 | M | 71 | 50 | Behavioural assessment not done | ECAS not done |
| ARTAG staining pattern | Glial | 3 | 0 | 0 | 1 | 2 | 1 | 1 |  |  |  |  |  |
| **Case 11** | Neuronal | 1 | 2 | 0 | 1 | 1 | 0 | 1 | M | 67 | 52 | Yes | No |
|  | Glial | 0 | 0 | 0 | 0 | 1 | 0 | 0 |  |  |  |  |  |
| **Case 12** | Neuronal | 3 | 0 | 1 | 1 | 1 | 1 | 1 | M | 71 | 24 | Behavioural assessment not done | Executive dysfunction |
|  | Glial | 1 | 0 | 1 | 1 | 1 | 1 | 1 |  |  |  |  |  |
| **Case 13** | Neuronal | 3 | 0 | 0 | 1 | 0 | 0 | 0 | F | 79 | 64 | No | No |
|  | Glial | 0 | 0 | 0 | 1 | 0 | 0 | 0 |  |  |  |  |  |
| **Case 14** | Neuronal | 3 | 3 | 2 | 2 | 2 | 3 | 1 | F | 67 | 60 | Behavioural assessment not done | ECAS not done |
|  | Glial | 0 | 2 | 1 | 1 | 2 | 2 | 2 |  |  |  |  |  |
| **Case 15** | Neuronal | 2 | 1 | 1 | 1 | 1 | 1 | 1 | F | 67 | 55 | Behavioural assessment not done | ECAS not done |
|  | Glial | 0 | 0 | 1 | 1 | 1 | 1 | 1 |  |  |  |  |  |
| **Case 16** | Neuronal | 1 | 0 | 0 | 0 | 0 | 0 | 0 | F | 45 | 54 | Behavioural assessment not done | No |
|  | Glial | 0 | 0 | 0 | 0 | 0 | 0 | 0 |  |  |  |  |  |
| **Case 17** | Neuronal | 3 | 1 | 0 | 0 | 0 | 0 | 0 | F | 76 | 11 | Behavioural assessment not done | ECAS not done |
| ARTAG staining pattern | Glial | 3 | 1 | 0 | 1 | 1 | 0 | 0 |  |  |  |  |  |
| **Case 18** | Neuronal | 3 | 1 | 0 | 0 | 0 | 0 | 0 | M | 69 | 16 | Yes | No |
| ARTAG staining pattern | Glial | 3 | 1 | 0 | 0 | 0 | 0 | 0 |  |  |  |  |  |
| **Case 19** | Neuronal | 1 | 0 | 0 | 1 | 3 | 1 | 1 | F | 58 | 24 | Behavioural assessment not done | ECAS not done |
|  | Glial | 0 | 0 | 0 | 1 | 2 | 0 | 0 |  |  |  |  |  |
| **Case 20** | Neuronal | 2 | 0 | 0 | 0 | 1 | 1 | 1 | M | 47 | 34 | Behavioural assessment not done | Language dysfunction |
|  | Glial | 0 | 0 | 0 | 1 | 1 | 1 | 1 |  |  |  |  |  |
| **Case 21** | Neuronal | 1 | 0 | 0 | 0 | 1 | 1 | 1 | M | 53 | 216 | Behavioural assessment not done | Language dysfunction |
|  | Glial | 0 | 0 | 0 | 1 | 1 | 1 | 1 |  |  |  |  |  |
| **Case 22** | Neuronal | 2 | 0 | 0 | 1 | 1 | 1 | 1 | M | 62 | 33 | Behavioural assessment not done | Language dysfunction |
|  | Glial | 0 | 0 | 0 | 1 | 1 | 1 | 1 |  |  |  |  |  |
| **Case 23** | Neuronal | 1 | 0 | 0 | 0 | 0 | 0 | 0 | F | 43 | 52 | Behavioural assessment not done | No |
|  | Glial | 0 | 0 | 0 | 0 | 0 | 0 | 0 |  |  |  |  |  |
| **Case 24** | Neuronal | 1 | 0 | 0 | 0 | 0 | 0 | 0 | M | 53 | 111 | No | No |
|  | Glial | 0 | 0 | 0 | 0 | 0 | 0 | 0 |  |  |  |  |  |
| **Case 25** | Neuronal | 1 | 0 | 0 | 0 | 1 | 0 | 0 | F | 55 | 36 | No | No |
|  | Glial | 0 | 0 | 0 | 0 | 1 | 0 | 0 |  |  |  |  |  |
| **Case 26** | Neuronal | 1 | 0 | 0 | 0 | 0 | 0 | 0 | M | 61 | 38 | Behavioural assessment not done | No |
|  | Glial | 0 | 0 | 0 | 0 | 0 | 0 | 0 |  |  |  |  |  |
| **Case 27** | Neuronal | 1 | 1 | 0 | 1 | 1 | 1 | 1 | M | 54 | 33 | Yes | Executive dysfunction |
|  | Glial | 0 | 1 | 1 | 1 | 2 | 1 | 1 |  |  |  |  |  |
| **Case 28** | Neuronal | 0 | 1 | 1 | 0 | 2 | 1 | 1 | M | 41 | 70 | Yes | No |
|  | Glial | 0 | 1 | 1 | 1 | 2 | 1 | 1 |  |  |  |  |  |
| **Case 29** | Neuronal | 1 | 3 | 2 | 1 | 3 | 1 | 1 | M | 69 | 48 | No | Fluency and executive dysfunction |
|  | Glial | 0 | 1 | 1 | 1 | 2 | 1 | 1 |  |  |  |  |  |
| **Case 30** | Neuronal | 1 | 0 | 0 | 0 | 0 | 0 | 0 | M | 56 | 44 | No | No |
|  | Glial | 0 | 0 | 0 | 0 | 0 | 0 | 0 |  |  |  |  |  |
